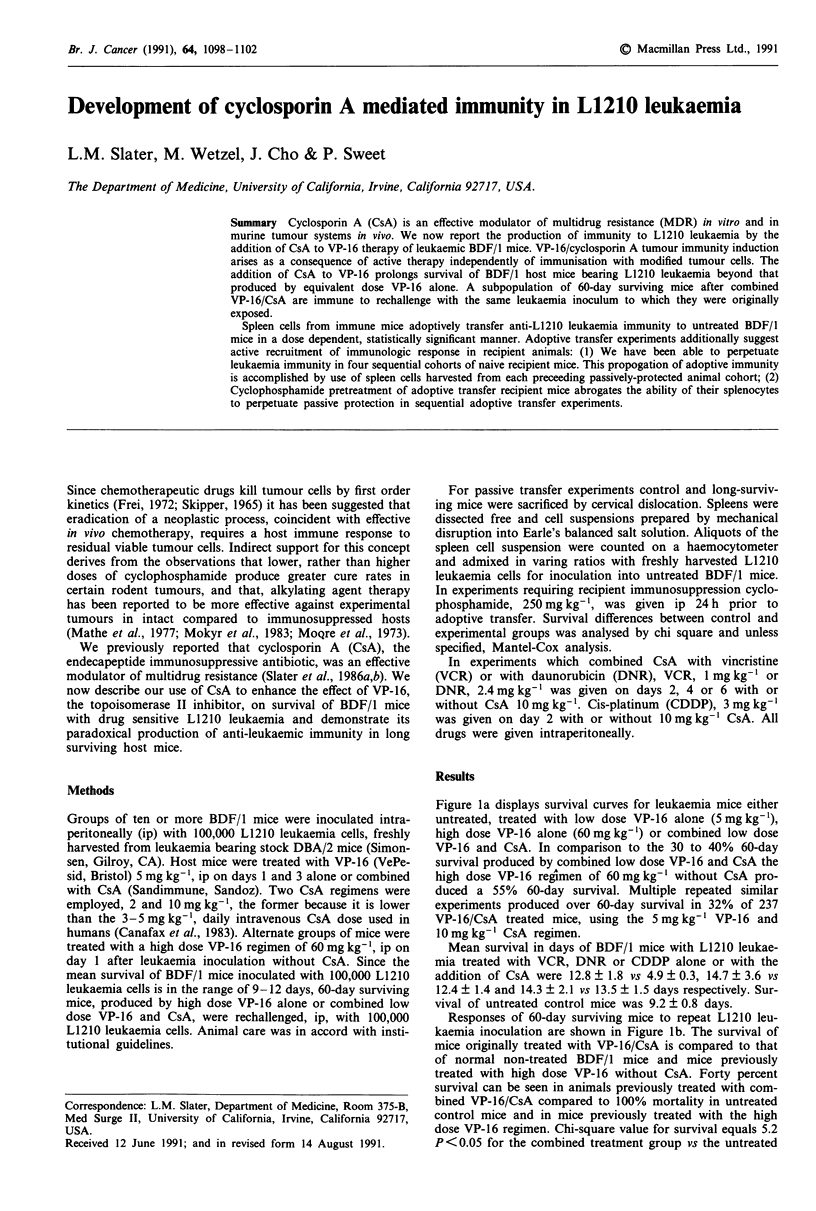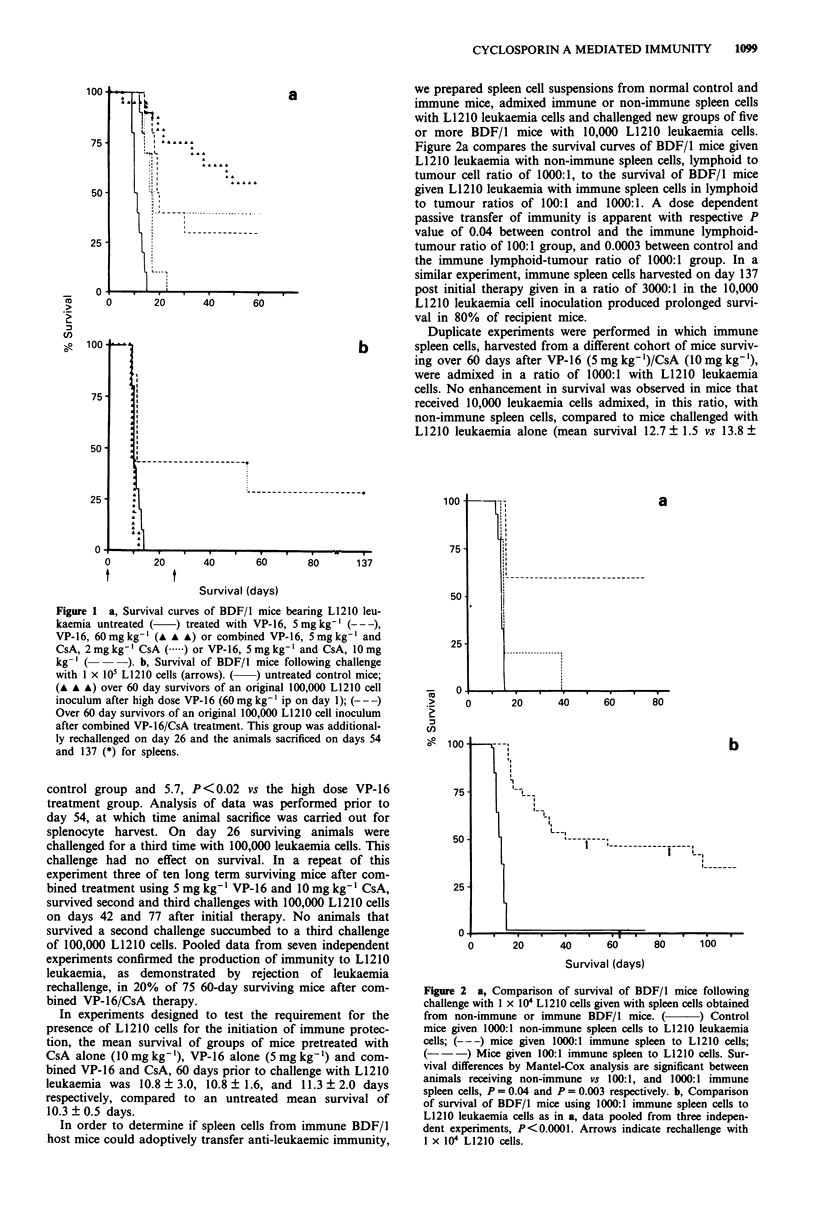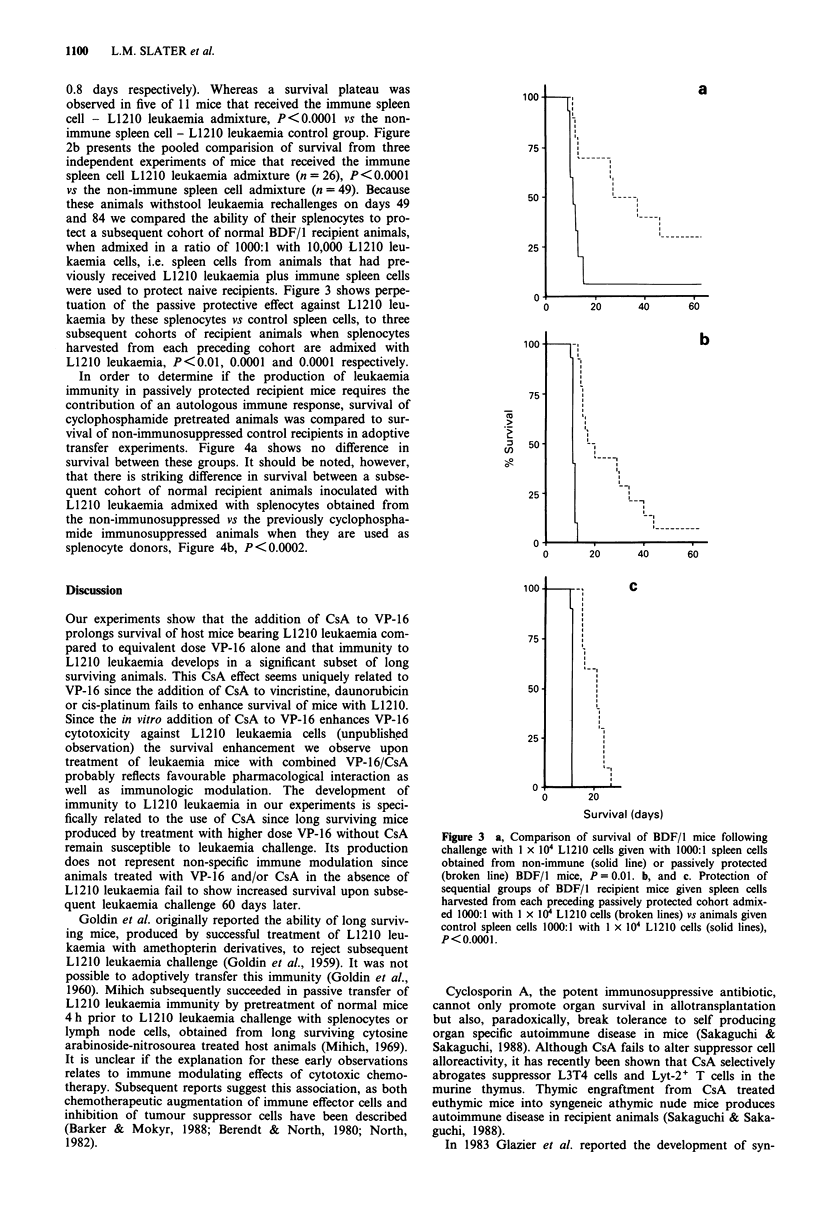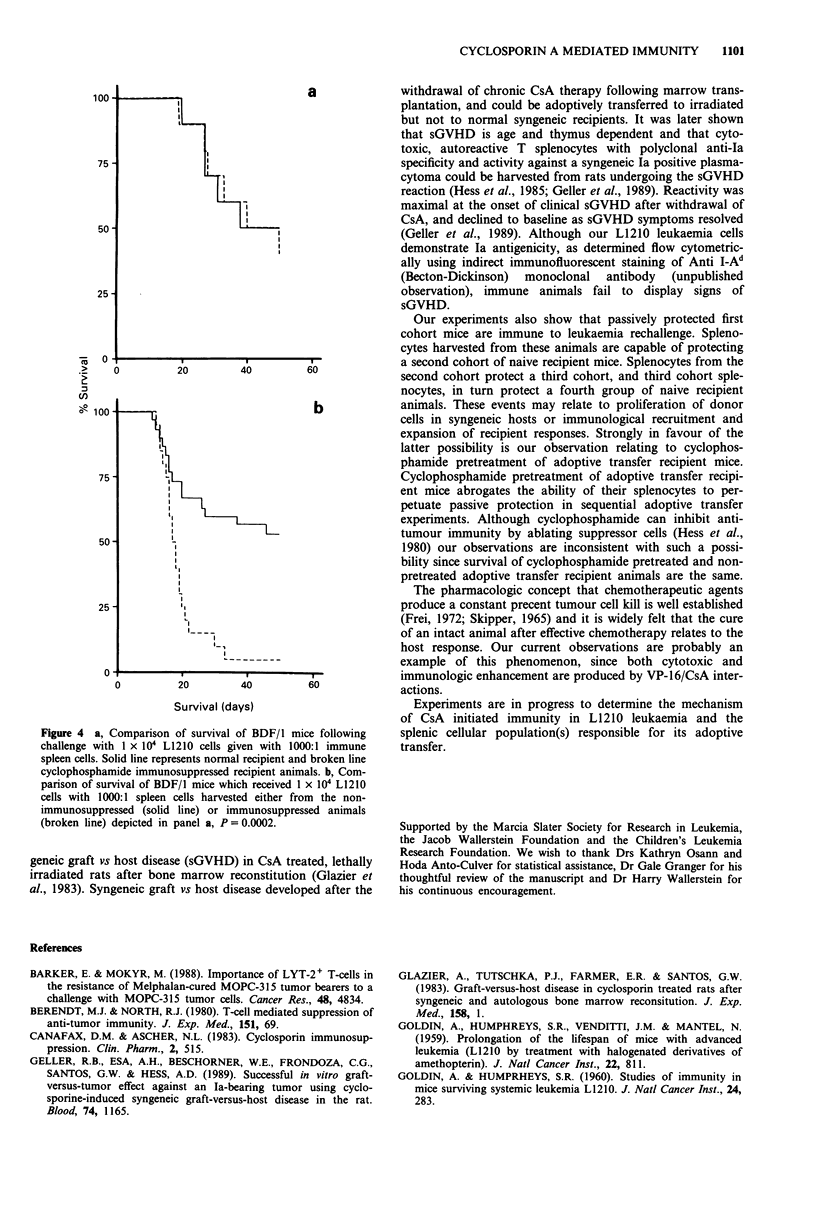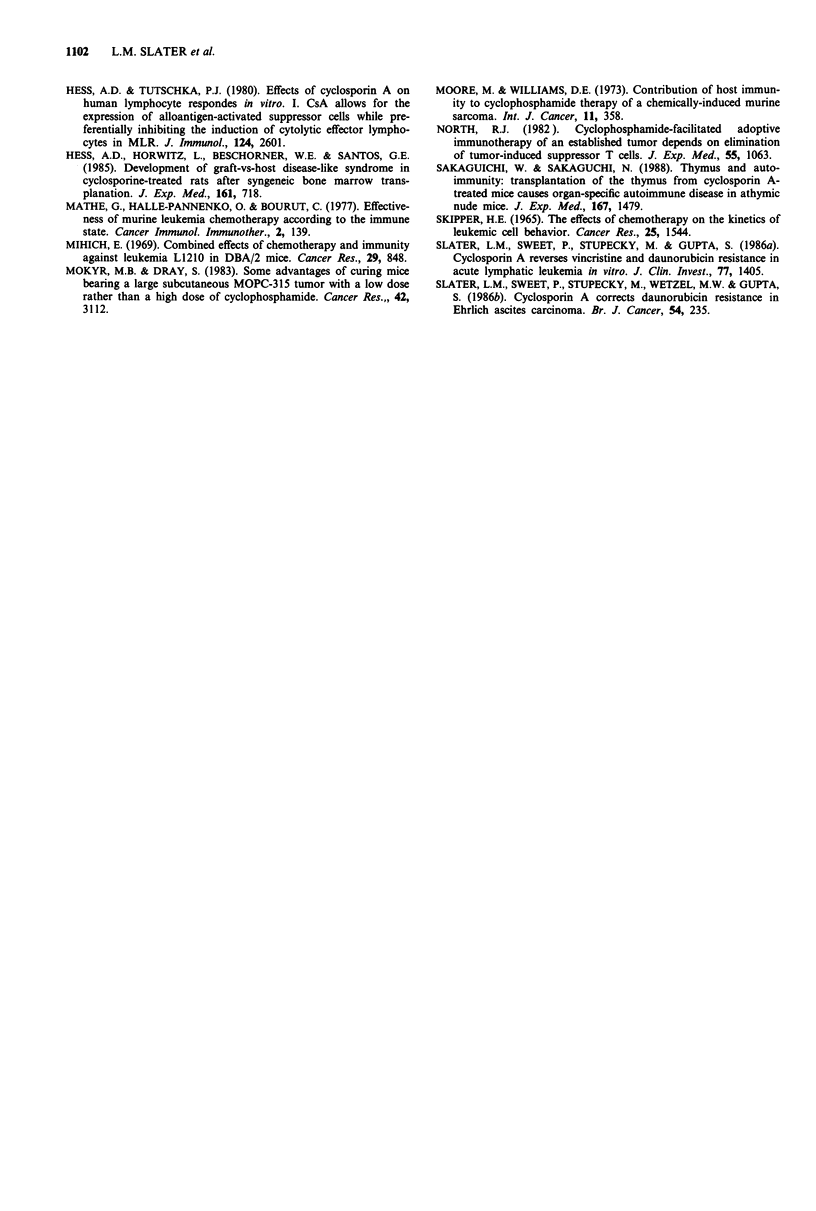# Development of cyclosporin A mediated immunity in L1210 leukaemia.

**DOI:** 10.1038/bjc.1991.471

**Published:** 1991-12

**Authors:** L. M. Slater, M. Wetzel, J. Cho, P. Sweet

**Affiliations:** Department of Medicine, University of California, Irvine 92717.

## Abstract

Cyclosporin A (CsA) is an effective modulator of multidrug resistance (MDR) in vitro and in murine tumour systems in vivo. We now report the production of immunity to L1210 leukaemia by the addition of CsA to VP-16 therapy of leukaemic BDF/1 mice. VP-16/cyclosporin A tumour immunity induction arises as a consequence of active therapy independently of immunisation with modified tumour cells. The addition of CsA to VP-16 prolongs survival of BDF/1 host mice bearing L1210 leukaemia beyond that produced by equivalent dose VP-16 alone. A subpopulation of 60-day surviving mice after combined VP-16/CsA are immune to rechallenge with the same leukaemia inoculum to which they were originally exposed. Spleen cells from immune mice adoptively transfer anti-L1210 leukaemia immunity to untreated BDF/1 mice in a dose dependent, statistically significant manner. Adoptive transfer experiments additionally suggest active recruitment of immunologic response in recipient animals: (1) We have been able to perpetuate leukaemia immunity in four sequential cohorts of naive recipient mice. This propogation of adoptive immunity is accomplished by use of spleen cells harvested from each preceeding passively-protected animal cohort; (2) Cyclophosphamide pretreatment of adoptive transfer recipient mice abrogates the ability of their splenocytes to perpetuate passive protection in sequential adoptive transfer experiments.


 © Macmillan Press Ltd., 1991

### Development of cyclosporin A mediated immunity in L1210 leukaemia

L.M. Slater, M. Wetzel, J. Cho & P. Sweet

*The Department of Medicine, University of California, Irvine, California 92717, USA.*

**Summary** Cyclosporin A (CsA) is an effective modulator of multidrug resistance (MDR) *in vitro* and in murine tumour systems *in vivo*. We now report the production of immunity to L1210 leukaemia by the addition of CsA to VP-16 therapy of leukaemic BDF/1 mice. VP-16/cyclosporin A tumour immunity induction arises as a consequence of active therapy independently of immunisation with modified tumour cells. The addition of CsA to VP-16 prolongs survival of BDF/1 host mice bearing L1210 leukaemia beyond that produced by equivalent dose VP-16 alone. A subpopulation of 60-day surviving mice after combined VP-16/CsA are immune to rechallenge with the same leukaemia inoculum to which they were originally exposed.

Spleen cells from immune mice adoptively transfer anti-L1210 leukaemia immunity to untreated BDF/1 mice in a dose dependent, statistically significant manner. Adoptive transfer experiments additionally suggest active recruitment of immunologic response in recipient animals: (1) We have been able to perpetuate leukaemia immunity in four sequential cohorts of naive recipient mice. This propogation of adoptive immunity is accomplished by use of spleen cells harvested from each preceeding passively-protected animal cohort; (2) Cyclophosphamide pretreatment of adoptive transfer recipient mice abrogates the ability of their splenocytes to perpetuate passive protection in sequential adoptive transfer experiments.

Since chemotherapeutic drugs kill tumour cells by first order kinetics (Frei, 1972; Skipper, 1965) it has been suggested that eradication of a neoplastic process, coincident with effective *in vivo* chemotherapy, requires a host immune response to residual viable tumour cells. Indirect support for this concept derives from the observations that lower, rather than higher doses of cyclophosphamide produce greater cure rates in certain rodent tumours, and that, alkylating agent therapy has been reported to be more effective against experimental tumours in intact compared to immunosuppressed hosts (Mathe *et al.*, 1977; Mokyr *et al.*, 1983; Moqre *et al.*, 1973).

We previously reported that cyclosporin A (CsA), the endecapeptide immunosuppressive antibiotic, was an effective modulator of multidrug resistance (Slater *et al.*, 1986*a*,*b*). We now describe our use of CsA to enhance the effect of VP-16, the topoisomerase II inhibitor, on survival of BDF/1 mice with drug sensitive L1210 leukaemia and demonstrate its paradoxical production of anti-leukaemic immunity in long surviving host mice.

### Methods

Groups of ten or more BDF/1 mice were inoculated intraperitoneally (ip) with 100,000 L1210 leukaemia cells, freshly harvested from leukaemia bearing stock DBA/2 mice (Simonsen, Gilroy, CA). Host mice were treated with VP-16 (VePesid, Bristol) 5 mg kg$^{-1}$, ip on days 1 and 3 alone or combined with CsA (Sandimmune, Sandoz). Two CsA regimens were employed, 2 and 10 mg kg$^{-1}$, the former because it is lower than the 3–5 mg kg$^{-1}$, daily intravenous CsA dose used in humans (Canafax *et al.*, 1983). Alternate groups of mice were treated with a high dose VP-16 regimen of 60 mg kg$^{-1}$, ip on day 1 after leukaemia inoculation without CsA. Since the mean survival of BDF/1 mice inoculated with 100,000 L1210 leukaemia cells is in the range of 9–12 days, 60-day surviving mice, produced by high dose VP-16 alone or combined low dose VP-16 and CsA, were rechallenged, ip, with 100,000 L1210 leukaemia cells. Animal care was in accord with institutional guidelines.

For passive transfer experiments control and long-surviving mice were sacrificed by cervical dislocation. Spleens were dissected free and cell suspensions prepared by mechanical disruption into Earle's balanced salt solution. Aliquots of the spleen cell suspension were counted on a haemocytometer and admixed in varing ratios with freshly harvested L1210 leukaemia cells for inoculation into untreated BDF/1 mice. In experiments requiring recipient immunosuppression cyclophosphamide, 250 mg kg$^{-1}$, was given ip 24 h prior to adoptive transfer. Survival differences between control and experimental groups was analysed by chi square and unless specified, Mantel-Cox analysis.

In experiments which combined CsA with vincristine (VCR) or with daunorubicin (DNR), VCR, 1 mg kg$^{-1}$ or DNR, 2.4 mg kg$^{-1}$ was given on days 2, 4 or 6 with or without CsA 10 mg kg$^{-1}$. Cis-platinum (CDDP), 3 mg kg$^{-1}$ was given on day 2 with or without 10 mg kg$^{-1}$ CsA. All drugs were given intraperitoneally.

### Results

Figure 1a displays survival curves for leukaemia mice either untreated, treated with low dose VP-16 alone (5 mg kg$^{-1}$), high dose VP-16 alone (60 mg kg$^{-1}$) or combined low dose VP-16 and CsA. In comparison to the 30 to 40% 60-day survival produced by combined low dose VP-16 and CsA the high dose VP-16 regimen of 60 mg kg$^{-1}$ without CsA produced a 55% 60-day survival. Multiple repeated similar experiments produced over 60-day survival in 32% of 237 VP-16/CsA treated mice, using the 5 mg kg$^{-1}$ VP-16 and 10 mg kg$^{-1}$ CsA regimen.

Mean survival in days of BDF/1 mice with L1210 leukaemia treated with VCR, DNR or CDDP alone or with the addition of CsA were 12.8 ± 1.8 *vs* 4.9 ± 0.3, 14.7 ± 3.6 *vs* 12.4 ± 1.4 and 14.3 ± 2.1 *vs* 13.5 ± 1.5 days respectively. Survival of untreated control mice was 9.2 ± 0.8 days.

Responses of 60-day surviving mice to repeat L1210 leukaemia inoculation are shown in Figure 1b. The survival of mice originally treated with VP-16/CsA is compared to that of normal non-treated BDF/1 mice and mice previously treated with high dose VP-16 without CsA. Forty percent survival can be seen in animals previously treated with combined VP-16/CsA compared to 100% mortality in untreated control mice and in mice previously treated with the high dose VP-16 regimen. Chi-square value for survival equals 5.2 $P<0.05$ for the combined treatment group *vs* the untreated

Correspondence: L.M. Slater, Department of Medicine, Room 375-B, Med Surge II, University of California, Irvine, California 92717, USA.
Received 12 June 1991; and in revised form 14 August 1991.

**Figure 1** **a**, Survival curves of BDF/1 mice bearing L1210 leukaemia untreated (——) treated with VP-16, 5 mg kg$^{-1}$ (- - -), VP-16, 60 mg kg$^{-1}$ (▲ ▲ ▲) or combined VP-16, 5 mg kg$^{-1}$ and CsA, 2 mg kg$^{-1}$ CsA (·····) or VP-16, 5 mg kg$^{-1}$ and CsA, 10 mg kg$^{-1}$ (— — —). **b**, Survival of BDF/1 mice following challenge with 1 × 10$^5$ L1210 cells (arrows). (——) untreated control mice; (▲ ▲ ▲) over 60 day survivors of an original 100,000 L1210 cell inoculum after high dose VP-16 (60 mg kg$^{-1}$ ip on day 1); (- - -) Over 60 day survivors of an original 100,000 L1210 cell inoculum after combined VP-16/CsA treatment. This group was additionally rechallenged on day 26 and the animals sacrificed on days 54 and 137 (*) for spleens.

control group and 5.7, $P<0.02$ *vs* the high dose VP-16 treatment group. Analysis of data was performed prior to day 54, at which time animal sacrifice was carried out for splenocyte harvest. On day 26 surviving animals were challenged for a third time with 100,000 leukaemia cells. This challenge had no effect on survival. In a repeat of this experiment three of ten long term surviving mice after combined treatment using 5 mg kg$^{-1}$ VP-16 and 10 mg kg$^{-1}$ CsA, survived second and third challenges with 100,000 L1210 cells on days 42 and 77 after initial therapy. No animals that survived a second challenge succumbed to a third challenge of 100,000 L1210 cells. Pooled data from seven independent experiments confirmed the production of immunity to L1210 leukaemia, as demonstrated by rejection of leukaemia rechallenge, in 20% of 75 60-day surviving mice after combined VP-16/CsA therapy.

In experiments designed to test the requirement for the presence of L1210 cells for the initiation of immune protection, the mean survival of groups of mice pretreated with CsA alone (10 mg kg$^{-1}$), VP-16 alone (5 mg kg$^{-1}$) and combined VP-16 and CsA, 60 days prior to challenge with L1210 leukaemia was 10.8 ± 3.0, 10.8 ± 1.6, and 11.3 ± 2.0 days respectively, compared to an untreated mean survival of 10.3 ± 0.5 days.

In order to determine if spleen cells from immune BDF/1 host mice could adoptively transfer anti-leukaemic immunity, we prepared spleen cell suspensions from normal control and immune mice, admixed immune or non-immune spleen cells with L1210 leukaemia cells and challenged new groups of five or more BDF/1 mice with 10,000 L1210 leukaemia cells. Figure 2a compares the survival curves of BDF/1 mice given L1210 leukaemia with non-immune spleen cells, lymphoid to tumour cell ratio of 1000:1, to the survival of BDF/1 mice given L1210 leukaemia with immune spleen cells in lymphoid to tumour ratios of 100:1 and 1000:1. A dose dependent passive transfer of immunity is apparent with respective *P* value of 0.04 between control and the immune lymphoid-tumour ratio of 100:1 group, and 0.0003 between control and the immune lymphoid-tumour ratio of 1000:1 group. In a similar experiment, immune spleen cells harvested on day 137 post initial therapy given in a ratio of 3000:1 in the 10,000 L1210 leukaemia cell inoculation produced prolonged survival in 80% of recipient mice.

Duplicate experiments were performed in which immune spleen cells, harvested from a different cohort of mice surviving over 60 days after VP-16 (5 mg kg$^{-1}$)/CsA (10 mg kg$^{-1}$), were admixed in a ratio of 1000:1 with L1210 leukaemia cells. No enhancement in survival was observed in mice that received 10,000 leukaemia cells admixed, in this ratio, with non-immune spleen cells, compared to mice challenged with L1210 leukaemia alone (mean survival 12.7 ± 1.5 *vs* 13.8 ±

**Figure 2** **a**, Comparison of survival of BDF/1 mice following challenge with 1 × 10$^4$ L1210 cells given with spleen cells obtained from non-immune or immune BDF/1 mice. (———) Control mice given 1000:1 non-immune spleen cells to L1210 leukaemia cells; (- - -) mice given 1000:1 immune spleen to L1210 cells; (— — —) Mice given 100:1 immune spleen to L1210 cells. Survival differences by Mantel-Cox analysis are significant between animals receiving non-immune *vs* 100:1, and 1000:1 immune spleen cells, $P = 0.04$ and $P = 0.003$ respectively. **b**, Comparison of survival of BDF/1 mice using 1000:1 immune spleen cells to L1210 leukaemia cells as in **a**, data pooled from three independent experiments, $P<0.0001$. Arrows indicate rechallenge with 1 × 10$^4$ L1210 cells.

0.8 days respectively). Whereas a survival plateau was observed in five of 11 mice that received the immune spleen cell – L1210 leukaemia admixture, $P<0.0001$ *vs* the non-immune spleen cell – L1210 leukaemia control group. Figure 2b presents the pooled comparision of survival from three independent experiments of mice that received the immune spleen cell L1210 leukaemia admixture ($n = 26$), $P<0.0001$ *vs* the non-immune spleen cell admixture ($n = 49$). Because these animals withstool leukaemia rechallenges on days 49 and 84 we compared the ability of their splenocytes to protect a subsequent cohort of normal BDF/1 recipient animals, when admixed in a ratio of 1000:1 with 10,000 L1210 leukaemia cells, i.e. spleen cells from animals that had previously received L1210 leukaemia plus immune spleen cells were used to protect naive recipients. Figure 3 shows perpetuation of the passive protective effect against L1210 leukaemia by these splenocytes *vs* control spleen cells, to three subsequent cohorts of recipient animals when splenocytes harvested from each preceding cohort are admixed with L1210 leukaemia, $P<0.01$, 0.0001 and 0.0001 respectively.

In order to determine if the production of leukaemia immunity in passively protected recipient mice requires the contribution of an autologous immune response, survival of cyclophosphamide pretreated animals was compared to survival of non-immunosuppressed control recipients in adoptive transfer experiments. Figure 4a shows no difference in survival between these groups. It should be noted, however, that there is striking difference in survival between a subsequent cohort of normal recipient animals inoculated with L1210 leukaemia admixed with splenocytes obtained from the non-immunosuppressed *vs* the previously cyclophosphamide immunosuppressed animals when they are used as splenocyte donors, Figure 4b, $P<0.0002$.

### Discussion

Our experiments show that the addition of CsA to VP-16 prolongs survival of host mice bearing L1210 leukaemia compared to equivalent dose VP-16 alone and that immunity to L1210 leukaemia develops in a significant subset of long surviving animals. This CsA effect seems uniquely related to VP-16 since the addition of CsA to vincristine, daunorubicin or cis-platinum fails to enhance survival of mice with L1210. Since the *in vitro* addition of CsA to VP-16 enhances VP-16 cytotoxicity against L1210 leukaemia cells (unpublished observation) the survival enhancement we observe upon treatment of leukaemia mice with combined VP-16/CsA probably reflects favourable pharmacological interaction as well as immunologic modulation. The development of immunity to L1210 leukaemia in our experiments is specifically related to the use of CsA since long surviving mice produced by treatment with higher dose VP-16 without CsA remain susceptible to leukaemia challenge. Its production does not represent non-specific immune modulation since animals treated with VP-16 and/or CsA in the absence of L1210 leukaemia fail to show increased survival upon subsequent leukaemia challenge 60 days later.

Goldin *et al.* originally reported the ability of long surviving mice, produced by successful treatment of L1210 leukaemia with amethopterin derivatives, to reject subsequent L1210 leukaemia challenge (Goldin *et al.*, 1959). It was not possible to adoptively transfer this immunity (Goldin *et al.*, 1960). Mihich subsequently succeeded in passive transfer of L1210 leukaemia immunity by pretreatment of normal mice 4 h prior to L1210 leukaemia challenge with splenocytes or lymph node cells, obtained from long surviving cytosine arabinoside-nitrosourea treated host animals (Mihich, 1969). It is unclear if the explanation for these early observations relates to immune modulating effects of cytotoxic chemotherapy. Subsequent reports suggest this association, as both chemotherapeutic augmentation of immune effector cells and inhibition of tumour suppressor cells have been described (Barker & Mokyr, 1988; Berendt & North, 1980; North, 1982).

**Figure 3** **a**, Comparison of survival of BDF/1 mice following challenge with $1 \times 10^4$ L1210 cells given with 1000:1 spleen cells obtained from non-immune (solid line) or passively protected (broken line) BDF/1 mice, $P = 0.01$. **b**, and **c**. Protection of sequential groups of BDF/1 recipient mice given spleen cells harvested from each preceding passively protected cohort admixed 1000:1 with $1 \times 10^4$ L1210 cells (broken lines) *vs* animals given control spleen cells 1000:1 with $1 \times 10^4$ L1210 cells (solid lines), $P<0.0001$.

Cyclosporin A, the potent immunosuppressive antibiotic, cannot only promote organ survival in allotransplantation but also, paradoxically, break tolerance to self producing organ specific autoimmune disease in mice (Sakaguchi & Sakaguchi, 1988). Although CsA fails to alter suppressor cell alloreactivity, it has recently been shown that CsA selectively abrogates suppressor L3T4 cells and Lyt-$2^+$ T cells in the murine thymus. Thymic engraftment from CsA treated euthymic mice into syngeneic athymic nude mice produces autoimmune disease in recipient animals (Sakaguchi & Sakaguchi, 1988).

In 1983 Glazier *et al.* reported the development of syn-

**Figure 4** **a**, Comparison of survival of BDF/1 mice following challenge with $1 \times 10^4$ L1210 cells given with 1000:1 immune spleen cells. Solid line represents normal recipient and broken line cyclophosphamide immunosuppressed recipient animals. **b**, Comparison of survival of BDF/1 mice which received $1 \times 10^4$ L1210 cells with 1000:1 spleen cells harvested either from the non-immunosuppressed (solid line) or immunosuppressed animals (broken line) depicted in panel **a**, $P = 0.0002$.

geneic graft *vs* host disease (sGVHD) in CsA treated, lethally irradiated rats after bone marrow reconstitution (Glazier *et al.*, 1983). Syngeneic graft *vs* host disease developed after the withdrawal of chronic CsA therapy following marrow transplantation, and could be adoptively transferred to irradiated but not to normal syngeneic recipients. It was later shown that sGVHD is age and thymus dependent and that cytotoxic, autoreactive T splenocytes with polyclonal anti-Ia specificity and activity against a syngeneic Ia positive plasmacytoma could be harvested from rats undergoing the sGVHD reaction (Hess *et al.*, 1985; Geller *et al.*, 1989). Reactivity was maximal at the onset of clinical sGVHD after withdrawal of CsA, and declined to baseline as sGVHD symptoms resolved (Geller *et al.*, 1989). Although our L1210 leukaemia cells demonstrate Ia antigenicity, as determined flow cytometrically using indirect immunofluorescent staining of Anti I-$A^d$ (Becton-Dickinson) monoclonal antibody (unpublished observation), immune animals fail to display signs of sGVHD.

Our experiments also show that passively protected first cohort mice are immune to leukaemia rechallenge. Splenocytes harvested from these animals are capable of protecting a second cohort of naive recipient mice. Splenocytes from the second cohort protect a third cohort, and third cohort splenocytes, in turn protect a fourth group of naive recipient animals. These events may relate to proliferation of donor cells in syngeneic hosts or immunological recruitment and expansion of recipient responses. Strongly in favour of the latter possibility is our observation relating to cyclophosphamide pretreatment of adoptive transfer recipient mice. Cyclophosphamide pretreatment of adoptive transfer recipient mice abrogates the ability of their splenocytes to perpetuate passive protection in sequential adoptive transfer experiments. Although cyclophosphamide can inhibit antitumour immunity by ablating suppressor cells (Hess *et al.*, 1980) our observations are inconsistent with such a possibility since survival of cyclophosphamide pretreated and non-pretreated adoptive transfer recipient animals are the same.

The pharmacologic concept that chemotherapeutic agents produce a constant precent tumour cell kill is well established (Frei, 1972; Skipper, 1965) and it is widely felt that the cure of an intact animal after effective chemotherapy relates to the host response. Our current observations are probably an example of this phenomenon, since both cytotoxic and immunologic enhancement are produced by VP-16/CsA interactions.

Experiments are in progress to determine the mechanism of CsA initiated immunity in L1210 leukaemia and the splenic cellular population(s) responsible for its adoptive transfer.

Supported by the Marcia Slater Society for Research in Leukemia, the Jacob Wallerstein Foundation and the Children's Leukemia Research Foundation. We wish to thank Drs Kathryn Osann and Hoda Anto-Culver for statistical assistance, Dr Gale Granger for his thoughtful review of the manuscript and Dr Harry Wallerstein for his continuous encouragement.

### References

BARKER, E. & MOKYR, M. (1988). Importance of LYT-$2^+$ T-cells in the resistance of Melphalan-cured MOPC-315 tumor bearers to a challenge with MOPC-315 tumor cells. *Cancer Res.*, **48**, 4834.

BERENDT, M.J. & NORTH, R.J. (1980). T-cell mediated suppression of anti-tumor immunity. *J. Exp. Med.*, **151**, 69.

CANAFAX, D.M. & ASCHER, N.L. (1983). Cyclosporin immunosuppression. *Clin. Pharm.*, **2**, 515.

GELLER, R.B., ESA, A.H., BESCHORNER, W.E., FRONDOZA, C.G., SANTOS, G.W. & HESS, A.D. (1989). Successful *in vitro* graft-versus-tumor effect against an Ia-bearing tumor using cyclosporine-induced syngeneic graft-versus-host disease in the rat. *Blood*, **74**, 1165.

GLAZIER, A., TUTSCHKA, P.J., FARMER, E.R. & SANTOS, G.W. (1983). Graft-versus-host disease in cyclosporin treated rats after syngeneic and autologous bone marrow reconsitution. *J. Exp. Med.*, **158**, 1.

GOLDIN, A., HUMPHREYS, S.R., VENDITTI, J.M. & MANTEL, N. (1959). Prolongation of the lifespan of mice with advanced leukemia (L1210 by treatment with halogenated derivatives of amethopterin). *J. Natl Cancer Inst.*, **22**, 811.

GOLDIN, A. & HUMPRHEYS, S.R. (1960). Studies of immunity in mice surviving systemic leukemia L1210. *J. Natl Cancer Inst.*, **24**, 283.

HESS, A.D. & TUTSCHKA, P.J. (1980). Effects of cyclosporin A on human lymphocyte respondes *in vitro*. I. CsA allows for the expression of alloantigen-activated suppressor cells while preferentially inhibiting the induction of cytolytic effector lymphocytes in MLR. *J. Immunol.*, **124**, 2601.

HESS, A.D., HORWITZ, L., BESCHORNER, W.E. & SANTOS, G.E. (1985). Development of graft-vs-host disease-like syndrome in cyclosporine-treated rats after syngeneic bone marrow transplanation. *J. Exp. Med.*, **161**, 718.

MATHE, G., HALLE-PANNENKO, O. & BOURUT, C. (1977). Effectiveness of murine leukemia chemotherapy according to the immune state. *Cancer Immunol. Immunother.*, **2**, 139.

MIHICH, E. (1969). Combined effects of chemotherapy and immunity against leukemia L1210 in DBA/2 mice. *Cancer Res.*, **29**, 848.

MOKYR, M.B. & DRAY, S. (1983). Some advantages of curing mice bearing a large subcutaneous MOPC-315 tumor with a low dose rather than a high dose of cyclophosphamide. *Cancer Res.,*, **42**, 3112.

MOORE, M. & WILLIAMS, D.E. (1973). Contribution of host immunity to cyclophosphamide therapy of a chemically-induced murine sarcoma. *Int. J. Cancer*, **11**, 358.

NORTH, R.J. (1982). Cyclophosphamide-facilitated adoptive immunotherapy of an established tumor depends on elimination of tumor-induced suppressor T cells. *J. Exp. Med.*, **55**, 1063.

SAKAGUICHI, W. & SAKAGUCHI, N. (1988). Thymus and autoimmunity: transplantation of the thymus from cyclosporin A-treated mice causes organ-specific autoimmune disease in athymic nude mice. *J. Exp. Med.*, **167**, 1479.

SKIPPER, H.E. (1965). The effects of chemotherapy on the kinetics of leukemic cell behavior. *Cancer Res.*, **25**, 1544.

SLATER, L.M., SWEET, P., STUPECKY, M. & GUPTA, S. (1986*a*). Cyclosporin A reverses vincristine and daunorubicin resistance in acute lymphatic leukemia *in vitro*. *J. Clin. Invest.*, **77**, 1405.

SLATER, L.M., SWEET, P., STUPECKY, M., WETZEL, M.W. & GUPTA, S. (1986*b*). Cyclosporin A corrects daunorubicin resistance in Ehrlich ascites carcinoma. *Br. J. Cancer*, **54**, 235.